# Low intensity extracorporeal shockwave therapy for chronic pelvic pain syndrome: Long-term follow-up

**DOI:** 10.1515/med-2023-0832

**Published:** 2023-10-28

**Authors:** Darijus Skaudickas, Povilas Lenčiauskas, Augustas Skaudickas, Greta Undžytė

**Affiliations:** Lithuania Medical Academy, Lithuanian University of Health Sciences, Eiveniu 2, 50161, Kaunas, Lithuania

**Keywords:** extracorporeal shock wave therapy, chronic pelvic pain, chronic non-bacterial prostatitis, shock waves

## Abstract

Chronic prostatitis (CP) is one of the diseases that reduce the quality of life (QoL) of young men. To date, there is no consensus on the management of these patients. It is essential to continue research into the treatment of CP, despite the use of various therapies, including low-energy extracorporeal shockwave therapy (ESWT). The main objective of this study is to observe and record the clinical symptomatology of patients during a 48-week follow-up period after ESWT treatment. Between 2019 and 2021, 28 patients with type IIIB CP/chronic pelvic pain syndrome were enrolled. Patients underwent ESWT once weekly for 4 weeks (3,000 individual sessions, maximum total energy flux density 0.25 mJ/mm^2^, frequency 3 Hz). Participants were assessed at 0, 4, 12, 24, 36 and 48 weeks post-treatment using the visual analogue scale (VAS), National Institutes of Health Chronic Prostatitis Symptom Index (NIH-CPSI), and International Index of Erectile Function (IIEF)-5. The mean age of patients was 47.1 ± 13.7 years (range 28–4 years). The positive effect of LI-ESWT was reflected in improvements in VAS, NIH-CPSI, and IIEF-5 scores. Regression of patients’ symptoms was observed as early as 4 weeks after treatment. The greatest progress was achieved at week 24. In addition, a slight worsening was observed at week 36 and 48, with stable progress. The treatment significantly improved the QoL of the patients, with the most significant improvement in the VAS score. In conclusion, this treatment approach is safe, most effective in the first 6 months. Thereafter, the efficacy of the treatment diminishes, but is sustained over a longer period.

## 1 Introduction

The chronic prostatitis (CP) is still a mystery, both diagnostically, clinically, and therapeutically. Many specialists, not only family doctors but also urologists, find it difficult to differentiate this pathology from other causes of small pelvic pain. A number of clinical studies have been carried out to prove the etiology and pathogenesis of pelvic pain syndrome. In recent years we have come to understand how vastly spread problem prostatitis is. The prevalence of prostatitis in the general population can get as high as 8.2% [1]. Meanwhile, another published study shows that these symptoms commonly affect younger males [2]. CP or chronic pelvic pain syndrome (CPPS) is the most common among all types of CP [3]. The quality of life (QoL) is substantially diminished due to CPPS symptoms, such as genitourinary pain, lower urinary tract symptoms, as well as sexual dysfunction and psychological disturbances that come along [4,5]. Research shows that erectile dysfunction affects most of the men with CP [6] and it is highly associated with very low mental-health scores in these patients [2,7]. Therefore, we notice a high increase in publications, especially on the topic of CPPS [8], which shows a need to further investigate the issue.

The etiology of CP/CPPS is still unknown. Even though it is unclear what exactly may cause this condition, researchers tend to believe that either yet unidentifiable infection or pelvic/genital trauma may lead the cause [9]. The primary suggestion behind deterioration of condition is inflammation and autoimmune response due to alterations in cytokine function [3,11]. Further research shows that interplay between endocrine and nervous system as well as central sensitization also play a big role in further development on chronicity of the symptoms [3,10,11]. Recently, more and more attention is being brought up to the psychological etiology aspect of CP/CPPS [12].

Referring to a classification system of prostatitis that has been found by NIH (1999), but renewed by European Association of Urology in 2018, prostatitis is divided into four distinct categories [13]. Out of the four categories, type III (chronic non-bacterial or CP/CPPS) is the most common, covering up to 90% of all cases of prostatitis [9]. Furthermore, the latter can be subdivided into categories IIIa (inflammatory) and IIIb (non-inflammatory), the main difference between them being evidence of white blood cells in the urine sample after the prostate massage (IIIa). In the literature another common term for IIIa prostatitis would be non-bacterial prostatitis, while for IIIb it is prostatodynia or abacterial prostatitis.

Although CP may seem to be a well-researched topic, the truth is that there is still a serious lack of more conclusive research data on the disease, especially on its treatment and its effectiveness. CP/CPPS is thought to be a heterogenous syndrome [14], in which management with monomodal therapy was highly unsuccessful [10]. Thus physicians use a wide variety of pharmacologic and nonpharmacologic treatment options to treat the condition. Research shows low intensity extracorporeal shock wave therapy (LI-ESWT) as a promising option for treatment [15]. However, more data should be collected on the effectiveness and long-term effects of LI-ESWT. Currently, there are only a few studies investigating the long-term (>12 months) efficacy of this treatment modality, and their conclusions are unclear or inconclusive. Therefore, the present work is a follow-up of a study conducted in 2020 [16], which followed up patients with long-term (>12 months) clinical data after extracorporeal lithotripsy treatment. This tactic of long-term follow-up and data collection up to 48 weeks apart has not been used before. Treatment using this approach is very interesting and important for us physicians because it is new, unexplored, and little published before. Knowing that LI-ESWT in the treatment of CPPS can be long-lasting would facilitate a comprehensive treatment tactic in patients with CP type IIIB/CPPS. Our working hypothesis was to investigate the efficacy and durability of this treatment approach.

## 2 Materials and methods

From 2019 to 2021, 28 patients with CP type IIIB/CPPS diagnosed based on the NIH International Prostatitis Collaboration Network report, referred to the Clinic of Urology, Lithuanian University of Health Sciences, were included in this study. A placebo group was not formed, knowing the effect of this treatment method on healthy men from past research works. All the patients provided signed informed consent. The Bioethics Centre of the Lithuanian University of Health Sciences granted approval to conduct this study (No. BEC-MF-335).

**Figure 1:**
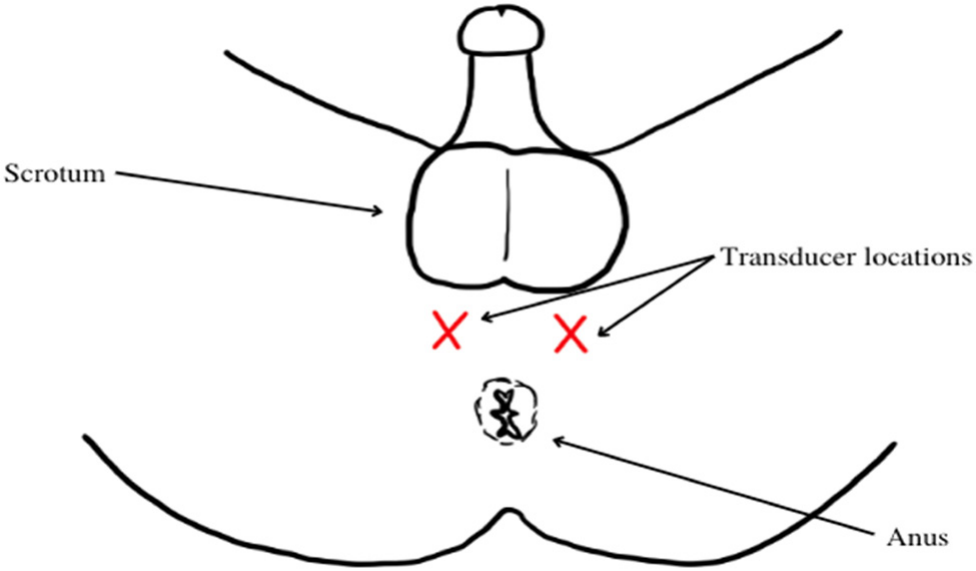
Transducer places during procedure.

Patients underwent ESWT once a week for 4 weeks (3,000 individually with a maximum total energy flow density of 0.25 mJ/mm^2^, rate 3 Hz). The appliance used to treat patients in this study was a typical electromagnetic shock wave device with a focused shock wave source (Duolith model SD1, Storz Medical, Switzerland). The location of the transducer was changed every 500 pulses perineally to affect the whole prostatic and pelvic floor region (Figure 1). Participants were evaluated at weeks 0, 4, 12, 24, 36, and 48 after treatment, using visual analogue scale (VAS), National Institutes of Health Chronic Prostatitis Symptom Index (NIH-CPSI), and International Index of Erectile Function (IIEF)-5. Two of the enrolled patients did not complete the study by failing to fill NIH-CPSI and IIEF-5 questionnaires at weeks 36 and 48. Data about patients prior treatment and prostate specific antigen levels were not gathered, therefore not discussed in this study. Given table concludes our research data. Data were compared using the paired-sample *t*-test. The statistical package IBM SPSS Statistics, version 21, was used for statistical analysis.

## 3 Results

The mean age of the patients was 47.1 ± 13.7 years (range 28–74). All the data presented in Table 1 have been proven to be statistically significant (except IIEF-5 questionnaire results of weeks 36 and 48). The table shows the general trend of symptoms improvement to be the most significant in week 0 – just after the end of treatment. Meanwhile, the extent of that improvement starts to reach clinical significance at weeks 12 and 24 with NIH-CPSI score deterioration at ≥678.57 and 89.29%, respectively. At week 24 we observed the biggest extent of positive changes (VAS 1.14 (±0.71), NIH-CPSI total 8.29 (±2.14), pain 3.00 (±0.98), urinary symptoms 2.11 (±0.83), QoL 3.18 (±1.02), IIEF-520.75 (±1.86)). Looking at further weeks’ results we can acknowledge that the effect of the treatment became less prominent than in the previous week as we see a slight deterioration in all symptom categories, although it is crucial to emphasize that this regression is quite minor. In comparison to the extent of symptoms and discomfort experienced by patients before treatment, long-term effects are clearly significant.

**Table 1:** Results: changes in parameters

Index	Prior to treatment	Week 0	Week 12	Week 24	Week 36	Week 48
VAS (±SD)	3.89 (±1.77)	2.61 (±1.40)	1.79* (±1.07)	1.14* (±0.71)	1.68* (±0.95)	1.71* (±1.08)
NIH-CPSI (±SD)						
Total	18.07 (±5.31)	13.89 (±4.50)	10.25* (±3.70)	8.29* (±2.14)	12.42* (±3.75)	12.62* (±3.93)
Pain	8.07 (±2.70)	6.04 (±2.12)	3.96* (±1.64)	3.00* (±0.98)	4.12* (±1.40)	4.42* (±1.53)
Urinary symptoms	4.32 (±1.91)	3.25 (±1.76)	2.61* (±1.32)	2.11* (±0.83)	3.23* (±1.82)	3.35* (±1.70)
QoL	5.68 (±1.87)	4.61 (±1.64)	3.68* (±1.28)	3.18* (±1.02)	5.08 (±1.32)	4.85* (±1.29)
NIH-CPSI deterioration ≥6, *n* (%)	—	8 (28.57)	22 (78.57)	25 (89.29)	10 (38.46)	13 (50.00)
IIEF-5 (±SD)	19.68 (±2.37)	19.89 (±2.11)	20.61* (±1.77)	20.75* (±1.86)	20.04 (±1.43)	20.04 (±1.48)

VAS, visual analogue scale; NIH-CPSI, National Institutes of Health Chronic Prostatitis Symptom Index; IIEF, the International Index of Erectile Function. Data marked by * indicates significance of *p* < 0.05.

Table 2 is aimed at showcasing the changes between two time periods in patients’ treatment. We have taken into account the comparison of week 0 and weeks 24 and 48 results. First thing that we can acknowledge is that mean differences as well as improvement are more substantial at week 24 than at week 48 in VAS, NIH-CPSI, and IIEF. The decline of this indicator suggests that there is a slight progression in patients’ symptoms. Also noticing the mean that most extensive changes are of NIH-CPSI total (9.79 and 5.46) and NIH-CPSI pain scores (5.07). Further examining the improvement of means we notice that most remarkable changes are of VAS score at week 24 (241.23%) and at week 48 (127.49%). Since pain reduction is the main criteria to determine patients QoL advancement, results suggest improved QoL. All data in this table are statistically significant (with the exception of NIH-CPIS QoL week 48 results).

**Table 2:** Results: changes in parameters

Parameter	95% CI	Mean difference	Improvement	*p* value
VAS (week 0)–VAS (week 24)	2.2–3.3	2.75	241.23	0.003
VAS (week 0)–VAS (week 48)	1.7–2.7	2.18	127.49	<0.001
NIH-CPSI (week 0)–NIH-CPSI (week 24)	8.1–11.5	9.79	117.97	<0.001
NIH-CPSI (week 0)–NIH-CPSI (week 48)	3.4–7.6	5.46	43.19	0.025
NIH-CPSI pain (week 0)–NIH-CPSI pain (week 24)	4.2–6.0	5.07	169	0.01
NIH-CPSI pain (week 0)–NIH-CPSI pain (week 48)	2.5–4.6	3.54	82.58	0.025
NIH-CPSI US (week 0)–NIH-CPSI US (week 24)	1.6–2.8	2.21	104.74	<0.001
NIH-CPSI US (week 0)–NIH-CPSI US (week 48)	0.2–1.8	1	28.96	0.019
NIH-CPSI QoL (week 0)–NIH-CPSI QoL (week 24)	1.9–3.2	2.5	78.62	0.014
NIH-CPSI QoL (week 0)–NIH-CPSI QoL (week 48)	0.1–1.8	0.92	17.11	0.259
IIEF-5 (week 0)–IIEF-5 (week 24)	−1.6 to −0.6	−1.07	−5.16	<0.001
IIEF-5 (week 0)–IIEF-5 (week 48)	−1.2 to 0.3	−0.42	−1.79	0.001

Data marked by * indicates significance of *p* < 0.05.

**Figure 2:**
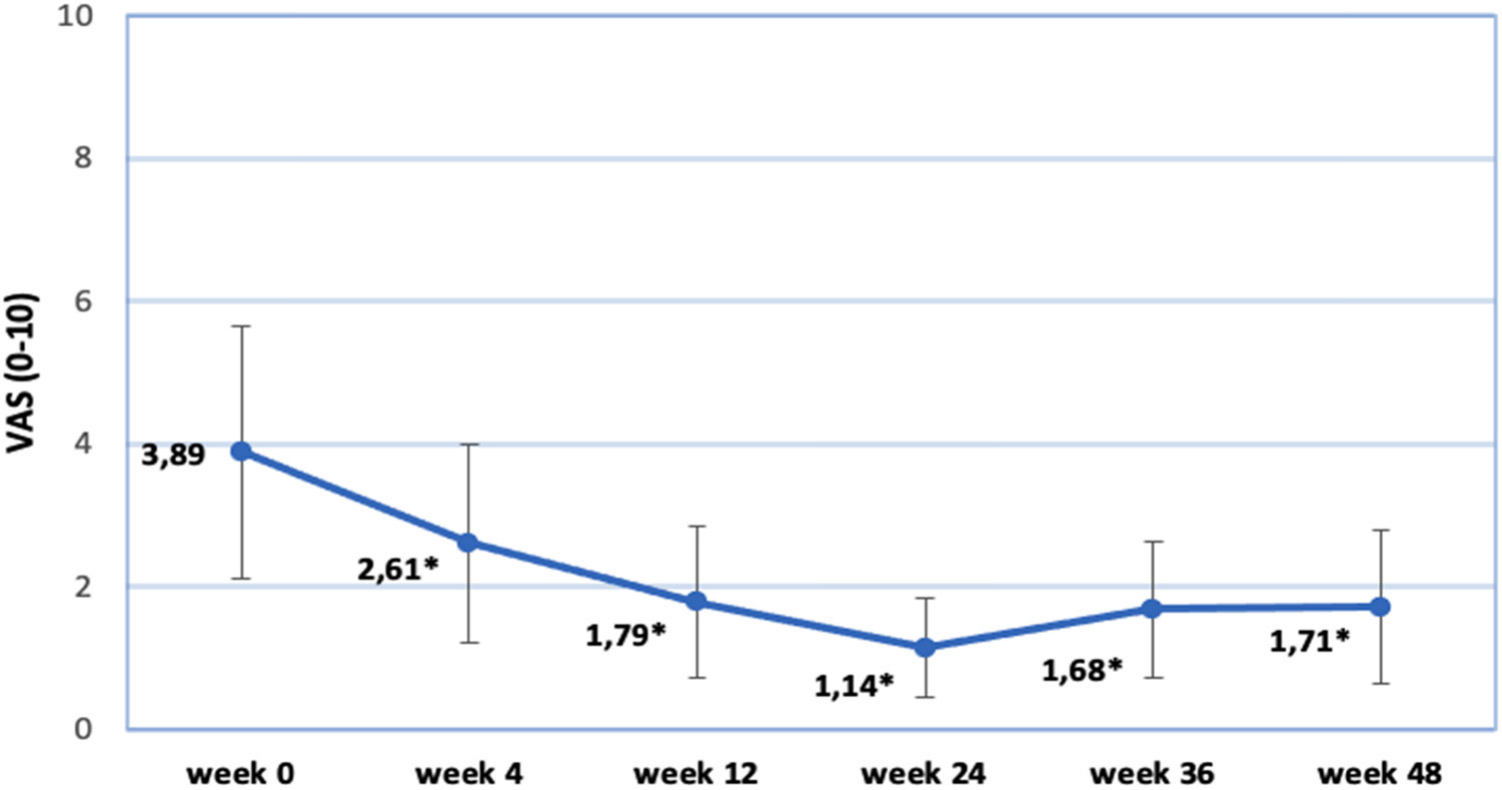
Changes in VAS score of pain.

Changes in the VAS score of pain are presented in Figure 2. Note that the most significant improvement is seen at comparing week 0 and week 4. Looking on a bigger scale, the results gradually advanced, reaching the peak at week 24 (1.14). Further on looking at weeks 36 and 48 we observe a slight deterioration in progress (1.68 and 1.71, respectively). All the results are statistically significant.

**Figure 3:**
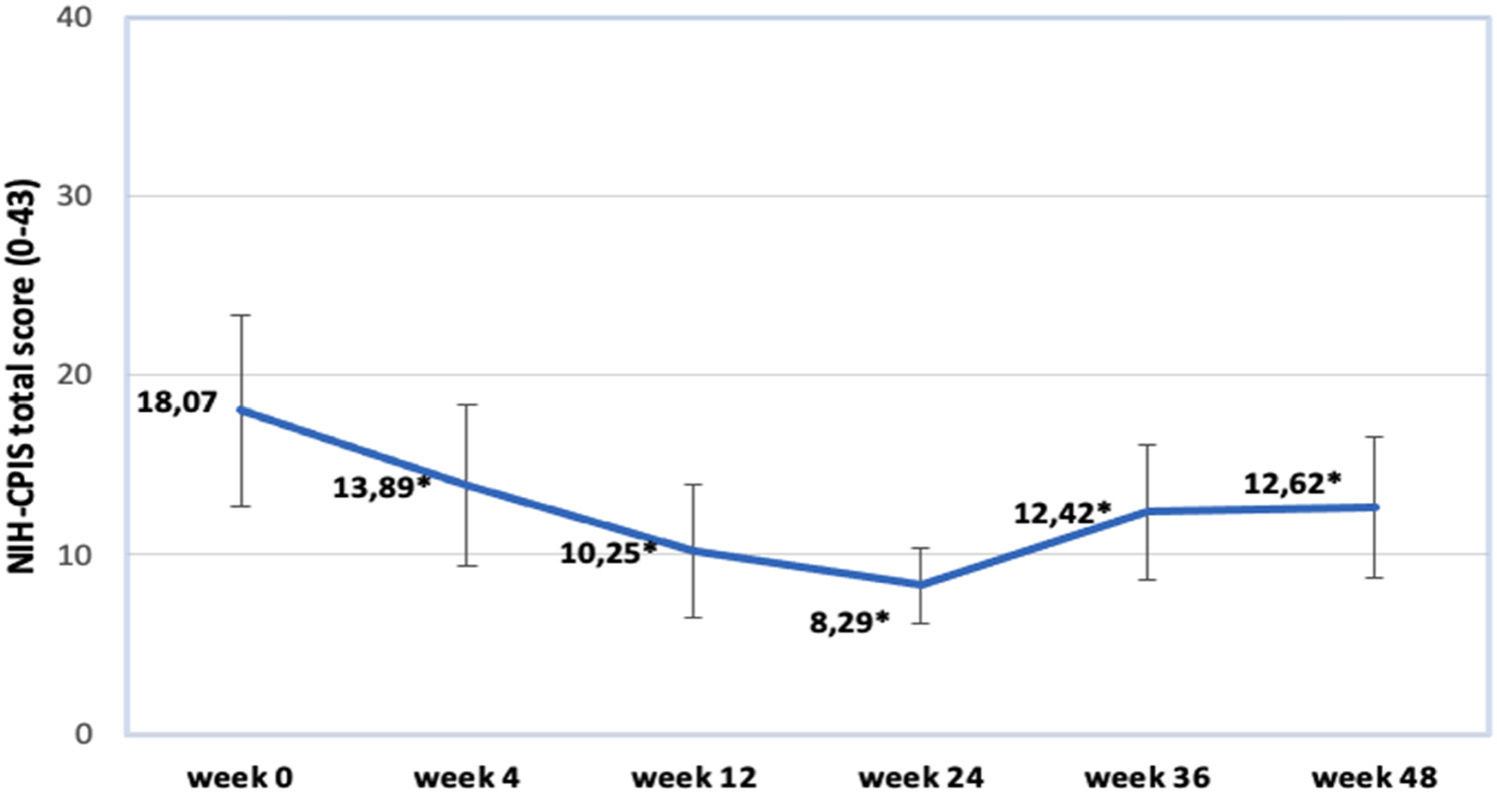
Changes in NIH-CPSI total score.

Changes in NIH-CPIS total score. Most significant improvement can be noticed comparing week 0 and week 4 results. Looking onward we see subtle advance in score results, reaching its pinnacle at week 24 (8.29). However, at weeks 36 and 48 we acknowledge a slight regression – scale results increase to 12.42 and 12.62, respectively. All the results are statistically significant. See Figure 3 for individual assessments of mentioned data.

**Figure 4:**
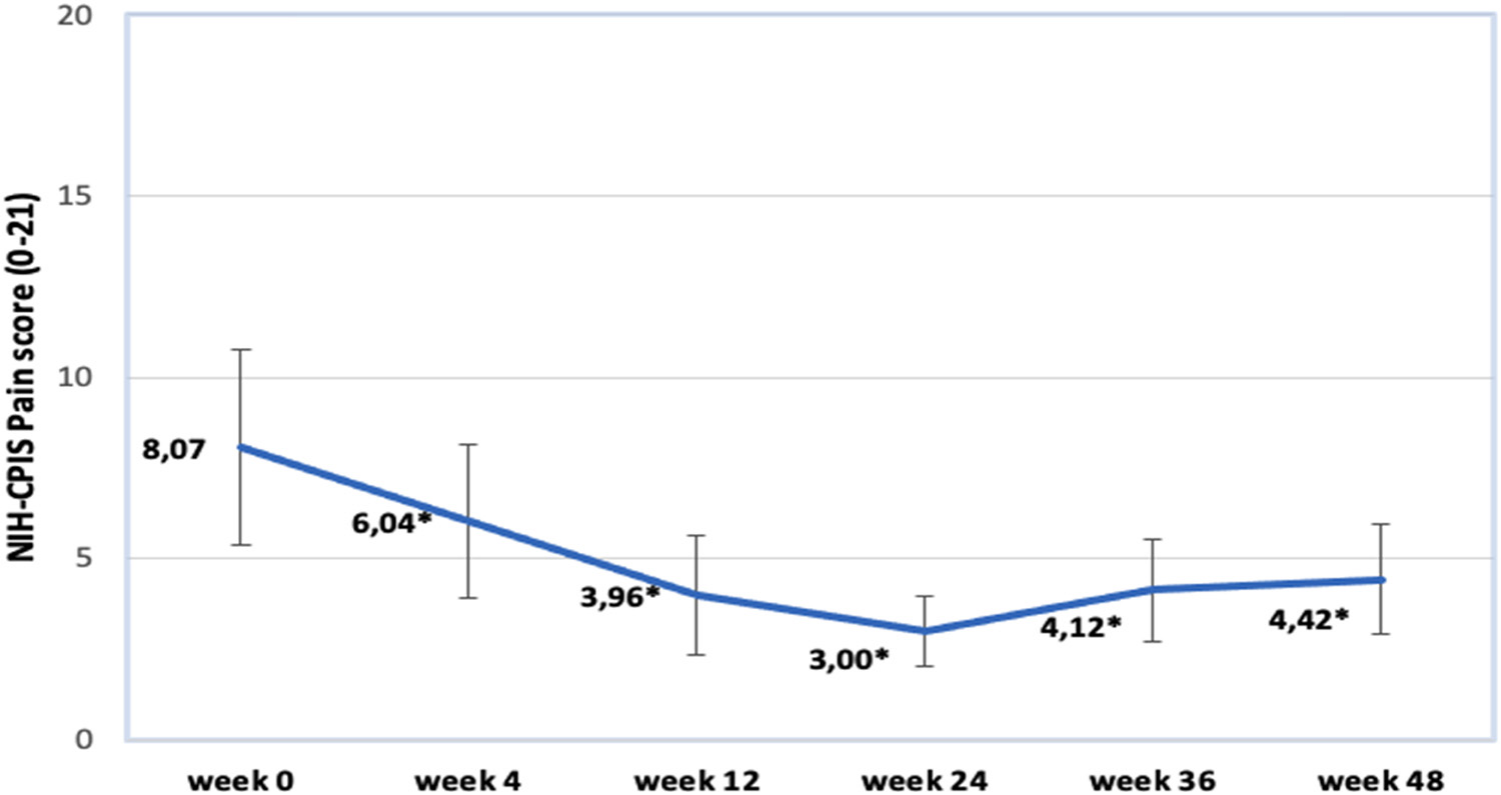
Changes in NIH-CPSI pain score. Data marked by * indicates significance of *p* < 0.05.

Looking at the changes in NIH-CPIS score we can see vast improvement at 4 and 12 weeks. Furthermore, advancements proceed until week 24 and reach its peak (3.00). Observing weeks 36 and 48 results we notice a minimal regression in the score results (4.12 and 4.42, respectively). All the results are statistically significant. This data further can be studied by looking at Figure 4.

**Figure 5:**
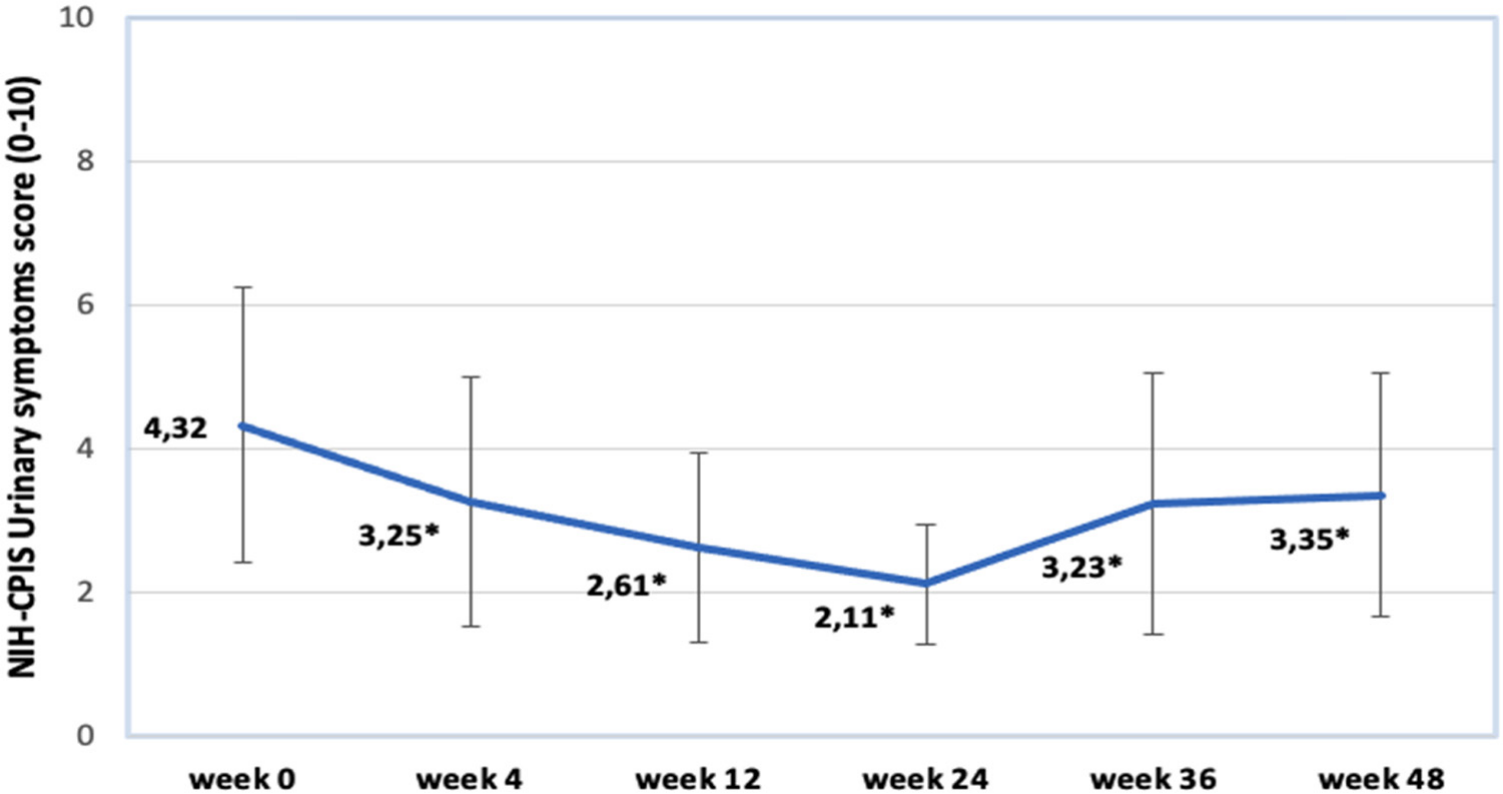
Changes in NIH-CPSI urinary score. Data marked by * indicates significance of *p* < 0.05.

Figure 5 shows changes in NIH-CPIS urinary symptoms score. The earliest improvement of urinary symptoms is observed comparing week 0 and week 4 results. The greatest extent of urinary symptoms regression is noticed at week 24 (2.11) and following that we see slight deterioration at weeks 36 and 48 (3.23 and 3.35). All the results are statistically significant.

**Figure 6:**
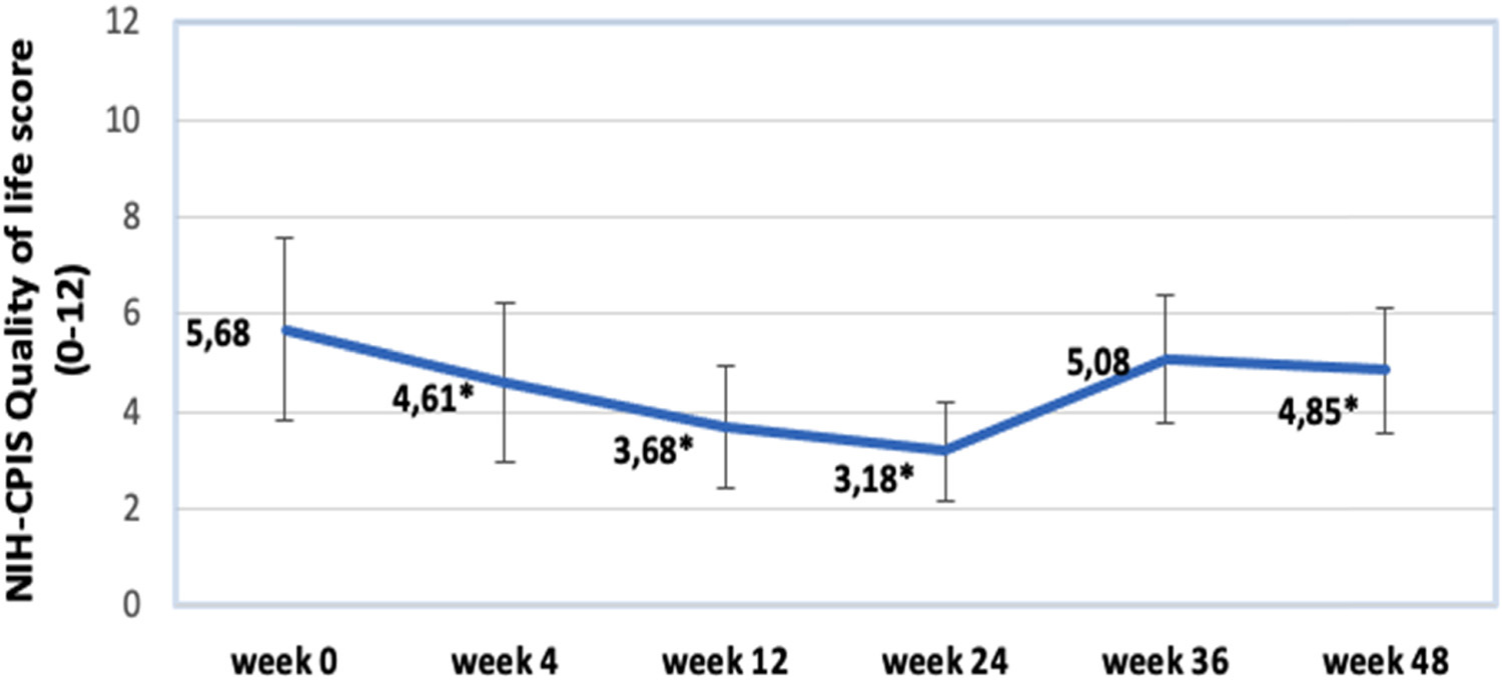
Changes in NIH-CPSI QoL score. Data marked by * indicates significance of *p* < 0.05.

Observing the changes in NIH-CPIS QoL score we can see the greatest improvement at week 24 (3.18). Meanwhile, in week 36 we notice a swift deterioration (5.08) in QoL, but at week 48 score also slightly advances further (4.85). All the results are statistically significant, except week 36 results. Summary of this data can be further analyzed in Figure 6.

**Figure 7:**
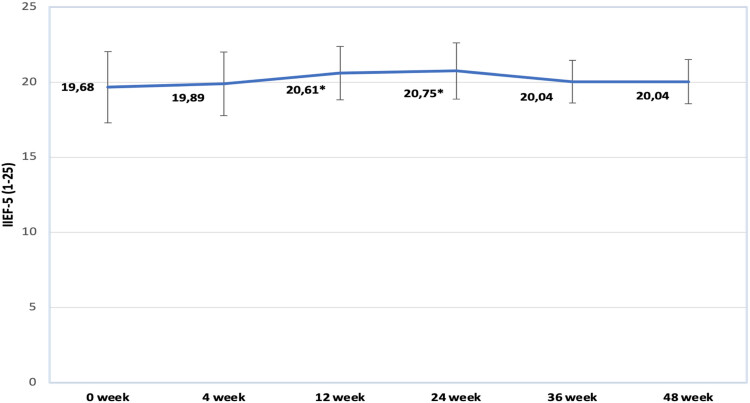
Changes in IIEF-5 score. Data marked by * indicates significance of *p* < 0.05.

Changes in IIEF-5 scores can be observed in Figure 7. Changes presented in the graph show that regression of ED symptoms is gradual, reaching its peak at week 24 (20.75). However, over the following weeks (36 and 48) we see a minor decline in score results (20.04).

**Figure 8:**
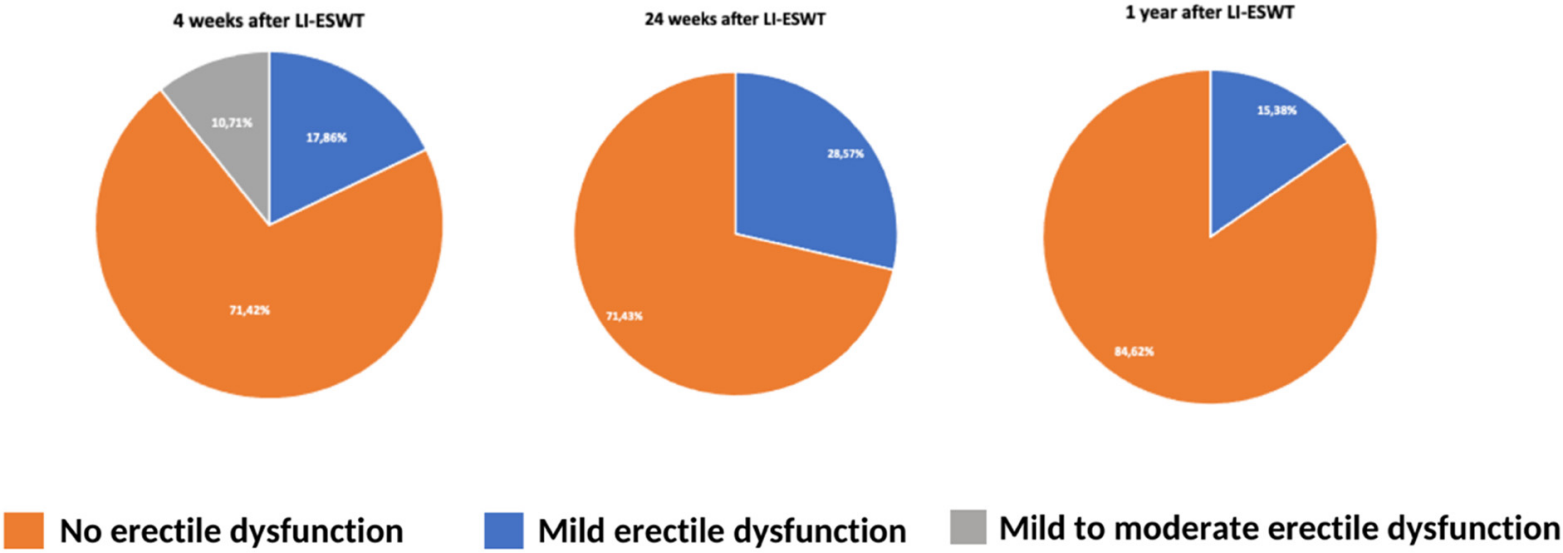
Changes in erectile dysfunction based on IIEF-5.

A series of pie charts analyzing changes in IIEF-5 scores throughout the treatment are shown in Figure 8. At week 4 we notice that there are a high number of patients experiencing mild to moderate erectile dysfunction symptoms (10.71%). Moving forward we acknowledge that at week 24 no more patients experience moderate ED symptoms, while the number of patients feeling mild symptoms grows (28.57%). Finally, 1 year after the treatment we can observe that most of the patients do not encounter ED (84.62%), while the ones who do only come across mild symptoms (15.38%).

## 4 Discussion

To this day, there is no uniformly accepted treatment regimen and the approach to each patient is tailored to his predominant symptoms. Since it is thought to be a heterogeneous syndrome, most case management consists of combined therapy, such as conventional 3-As therapy [17]. The latter is a combination of antibiotics, non-steroid anti-inflammatory agents, and alpha blockers. Furthermore, pharmacologic therapy can be expanded using 5-alpha-reductase inhibitors, neuromodulators, or homeopathic drugs [18]. The complex treatment of this disease also incorporate non-pharmacologic therapies, such as acupuncture, transrectal thermotherapy, physical therapy of pelvic floor muscles, LI-ESWT, prostate massage, and botulinum toxin A intraprostatic injections [18,19]. Due to such variety and complexity in treatment, in many cases the condition prolongs the impairment of QoL (not only for the patient, but also for their partners) [3].

ESWT – a method that has been introduced more than 20 years ago, to fragment kidney stones, now has been receiving a wide application in many fields of medicine. Due to its angiogenesis inducing effect LI-ESWT is one of the most promising non-invasive treatment options for different types of tendinopathies, sports injuries [20,21] as well as ischemic cardiac disease [22,23]. More ways of use have been discovered in the field of urology. Now it is seen as a prospective treatment method of erectile dysfunction [24] and CPSS [25]. Even though the precise molecular mechanism is not clear, it is suspected that increased production and secretion of nitric oxide and endothelial vascular growth factor may be responsible for induced angiogenesis [23,26]. Meanwhile, other researchers suggest that intracellular regeneration [27] and COX-2 inhibition [28] may cause anti-inflammatory effects. Besides that, the mechanism behind pain reduction could also be explained by overstimulation and regeneration of nerve fibers [29].

In the first study Zimmermann et al. [30] laid the groundwork for further research of LI-ESWT. This research has provided evidence that the latter is a promising method for treatment of CPPS. The overall pain reduction rate in patients after week 12 treatment was 44%. Furthermore, NIH-CPIS scores have improved by 22.5% for 10 out of 14 patients. In 2009 research on the same topic Zimmermann et al. [25] showed that usage of LI-ESWT provided a significant improvement of pain, urinary symptoms, and QoL for all enrolled patients.

Guu et al. [31] researched the effectiveness of LI-ESWT to patients who had been receiving 3-A medicine treatment for the last 6 weeks. The authors concluded that at week 4 after treatment 27 of 33 (81.82%) patients had a positive response to therapy. Great improvement to the VAS score was observed. Since it is the main parameter for the effectiveness of treating CPSS and enhancement of QoL, researchers concluded that treatment was effective.

In systematic review and meta-analysis by Kong et al. [32] effectiveness of LI-ESWT procedure alone was compared to treatment, combined with medication. Research concludes that combined treatment LI-ESWT and medications had a more significant and longer lasting improvement in VAS, IIEF-5, and IPSS scores.

In another study Li and Man [33] has exhibited substantial improvement of VAS and NIH-CPSI scores at week 4 after the treatment of LI-ESWT, which lasted up to week 12. Therefore the treatment’s short-term effectiveness was recognized. Authors of this research state that the procedure has not caused any side effects to the patients. Compared to our study, our patients have not experienced any side effects related to the treatment and short-term improvement in VAS and NIH-CPSI was observed as well.

Al Edwan et al. [34] were the first to study long-term efficacy of LI-ESWT for CPSS. According to the research week 2 after treatment improvement in NIH-CPSI urinary symptoms score (38%) and AUA QoL_US score (38%) was observed. The most substantial progress has been achieved at 6 months after treatment. Furthermore, this improvement of symptoms was maintained for 12 months (with a slight decrease). These conclusions concur with our research data that also show an improvement of greatest extent at week 24 and long lasting (>12 months) positive effects on CPSS symptoms compared to week 0.

In another study by Guu et al. [17] 83.9% of the patients who experienced short-term effects, continued therapeutic efficacy for 12 months. The positive effects were observed across IPSS, VAS, and NIH-CPSI total scores. Meanwhile, Mykoniatis et al. [35] in their meta-analysis study came to a conclusion that long-term effects of LI-ESWT are clinically insignificant.

In our study a positive impact of the LI-ESWT procedure was observed as improvement in VAS, NIH-CPSI. The regression of patient’s symptoms was noticeable after 4 weeks of therapy. The greatest extent of progress had been achieved at week 24. Furthermore, at weeks 36 and 48 we have observed a slight deterioration, but while still retaining stable progress. While observing changes in IIEF-5 score we notice similar tendencies of improvement, with effectiveness reaching its peak at 48 weeks, although we have to note that IIEF-5 score results of weeks 36 and 48 were not statistically significant. In the end, patients’ QoL has been improved significantly by the treatment, since the greatest improvement was in VAS score.

## 5 Conclusions

We can conclude that LI-ESWT is an effective therapy for CPSS. Furthermore, this method has proven to be safe for the patient and repeatable at any given time during the whole treatment. However, there still is a great demand of more long term and bigger scale studies on LI-ESWT procedures efficacy comparing to current CPSS treatment guidelines.

## References

[1] Krieger JN, Lee SW, Jeon J, Cheah PY, Liong ML, Riley DE. Epidemiology of prostatitis. Int J Antimicrob Agents. 2008 Feb;31(Suppl 1):S85–90. 10.1016/j.ijantimicag.2007.08.028. Epub 2007. Dec 31 PMID: 18164907; PMCID: PMC2292121. PMC2292121 18164907

[2] Sugimoto M, Hijikata Y, Tohi Y, Kuroda H, Takei M, Matsuki T, et al. Low quality of life in men with chronic prostatitis-like symptoms. Prostate Cancer Prostatic Dis. 2022 Apr;25(4):785–90. 10.1038/s41391-022-00559-w. Epub 2022 Jun 25. PMID: 35752656; PMCID: PMC9705241. PMC9705241 35752656

[3] Pontari MA, Ruggieri MR. Mechanisms in prostatitis/chronic pelvic pain syndrome. J Urol. 2004 Sep;172(3):839–45. 10.1097/01.ju.0000136002.76898.04. PMID: 15310980; PMCID: PMC3591463. PMC3591463 15310980

[4] Mändar R, Korrovits P, Rahu K, Rahu M, Sibul EL, Mehik A, et al. Dramatically deteriorated quality of life in men with prostatitis-like symptoms. Andrology. 2020 Jan;8(1):101–9. 10.1111/andr.12647. Epub 2019 May 15. PMID: 31090261. 31090261

[5] Vahdatpour B, Alizadeh F, Moayednia A, Emadi M, Khorami MH, Haghdani S. Efficacy of extracorporeal shock wave therapy for the treatment of chronic pelvic pain syndrome: a randomized, controlled trial. ISRN Urol. 2013 Aug;2013:972601. 10.1155/2013/972601. PMID: 24000311; PMCID: PMC3755541. PMC3755541 24000311

[6] Daneshwar D, Nordin A. Low intensity extracorporeal shockwave therapy for chronic pelvic pain syndrome patients with erectile dysfunction. Medicine. 2022 Jan;101(2):e28546. 10.1097/MD.0000000000028546. PMC8758023 35029213

[7] Peciulyte G, Jonusiene G, Skaudickas D. PS-01-017 evaluation of personality traits, sexual function, and prevalence of masturbation in men with chronic prostatitis/chronic pelvic pain syndrome. J Sex Med. 2016;13(5):S81–2.

[8] Chen Q, Feng J, Liu Z, An D, Li Y, Zhou S, et al. Research trends of prostatitis over past 20 years: a bibliometric analysis. Andrologia. 2021 Nov;53(10):e14206. 10.1111/and.14206 34365673

[9] Zaidi N, Thomas D, Chughtai B. Management of chronic prostatitis. Curr Urol Rep. 2018 Aug;19(11):88. 10.1007/s11934-018-0841-9. PMID: 30167899. 30167899

[10] Pena VN, Engel N, Gabrielson AT, Rabinowitz MJ, Herati AS. Diagnostic and management strategies for patients with chronic prostatitis and chronic pelvic pain syndrome. Drugs Aging. 2021;38:845–86. 10.1007/s40266-021-00890-2. 34586623

[11] Pontari MA. Etiology of chronic prostatitis/chronic pelvic pain syndrome: psychoimmunoneurendocrine dysfunction (PINE syndrome) or just a really bad infection? World J Urol. 2013 Aug;31(4):725–32. 10.1007/s00345-013-1061-z. Epub 2013 Apr 12. PMID: 23579440. 23579440

[12] Riegel B, Bruenahl CA, Ahyai S, Bingel U, Fisch M, Löwe B. Assessing psychological factors, social aspects and psychiatric co-morbidity associated with Chronic Prostatitis/Chronic Pelvic Pain Syndrome (CP/CPPS) in men – a systematic review. J Psychosom Res. 2014 Nov;77(5):333–50. 10.1016/j.jpsychores.2014.09.012. Epub 2014 Sep 30. PMID: 25300538. 25300538

[13] Bonkat G, Pickard R, Bartoletti R, Cai T, BruyÃ©re F, Geerlings SE, et al. EAU guidelines on urological infections 2018. European Association of Urology Guidelines. 2018 Edition. Arnhem, The Netherlands: European Association of Urology Guidelines Office; 2018.

[14] Polackwich AS, Shoskes DA. Chronic prostatitis/chronic pelvic pain syndrome: a review of evaluation and therapy. Prostate Cancer Prostatic Dis. 2016 Jun;19(2):132–8. 10.1038/pcan.2016.8. Epub 2016 Mar 8. PMID: 26951713. 26951713

[15] Moayednia A, Haghdani S, Khosrawi S, Yousefi E, Vahdatpour B. Long-term effect of extracorporeal shock wave therapy on the treatment of chronic pelvic pain syndrome due to non bacterial prostatitis. J Res Med Sci. 2014 Apr;19(4):293–6. PMID: 25097599; PMCID: PMC4115342. PMC4115342 25097599

[16] Skaudickas D, Telksnys T, Veikutis V, Aniulis P, Jievaltas M. Extracorporeal shock wave therapy for the treatment of chronic pelvic pain syndrome. Open Med (Wars). 2020 Jul;15(1):580–5. 10.1515/med-2020-0174. PMID: 33336014; PMCID: PMC7712093. PMC7712093 33336014

[17] Guu SJ, Liu CC, Juan YS, Li CC, Tsai CC. The 12-month follow-up of the low-intensity extracorporeal shockwave therapy in the treatment of patients with chronic pelvic pain syndrome refractory to 3-As medications. The Aging Male. 2019;23(5):1–8. 10.1080/13685538.2019.1597341 30945953

[18] Franco JVA, Turk T, Jung JH, Xiao YT, Iakhno S, Tirapegui FI, et al. Pharmacological interventions for treating chronic prostatitis/chronic pelvic pain syndrome: a Cochrane systematic review. BJU Int. 2020;125(4):490–6. 10.1111/bju.14988 31899937

[19] Franco JV, Turk T, Jung JH, Xiao YT, Iakhno S, Garrote V, et al. Non-pharmacological interventions for treating chronic prostatitis/chronic pelvic pain syndrome. Cochrane Database Syst Rev. 2018 May;5(5):CD012551. 10.1002/14651858.CD012551.pub3. PMID: 29757454; PMCID: PMC6494451. PMC6494451 29757454

[20] Gatz M, Schweda S, Betsch M, Dirrichs T, de la Fuente M, Reinhardt N, et al. Line- and point-focused extracorporeal shock wave therapy for achilles tendinopathy: a placebo-controlled RCT study. Sports Health. 2021 Sep–Oct;13(5):511–8. 10.1177/1941738121991791. Epub 2021 Feb 13. PMID: 33586526; PMCID: PMC8404720. PMC8404720 33586526

[21] Schroeder AN, Tenforde AS, Jelsing EJ. Extracorporeal shockwave therapy in the management of sports medicine injuries. Curr Sports Med Rep. 2021 Jun;20(6):298–305. 10.1249/JSR.0000000000000851. PMID: 34099607. 34099607

[22] Ito K, Fukumoto Y, Shimokawa H. Extracorporeal shock wave therapy for ischemic cardiovascular disorders. Am J Cardiovasc Drugs. 2011 Oct;11(5):295–302. 10.2165/11592760-000000000-00000. PMID: 21846155. 21846155

[23] Yang HT, Xie X, Hou XG, Xiu WJ, Wu TT. Cardiac shock wave therapy for coronary heart disease: an updated meta-analysis. Braz J Cardiovasc Surg. 2020 Oct;35(5):741–56. 10.21470/1678-9741-2019-0276. PMID: 33118740; PMCID: PMC7598952. PMC7598952 33118740

[24] Rizk PJ, Krieger JR, Kohn TP, Pastuszak AW. Low-intensity shockwave therapy for erectile dysfunction. Sex Med Rev. 2018 Oct;6(4):624–30. 10.1016/j.sxmr.2018.01.002. Epub 2018 Mar 22. PMID: 29576441. 29576441

[25] Zimmermann R, Cumpanas A, Miclea F, Janetschek G. Extracorporeal shock wave therapy for the treatment of chronic pelvic pain syndrome in males: a randomised, double-blind, placebo-controlled study. Eur Urol. 2009 Sep;56(3):418–24. 10.1016/j.eururo.2009.03.043. Epub 2009 Mar 25 Erratum in: Eur Urol. 2020 May;77(5):e140. PMID: 19372000. 19372000

[26] Mariotto S, de Prati AC, Cavalieri E, Amelio E, Marlinghaus E, Suzuki H. Extracorporeal shock wave therapy in inflammatory diseases: molecular mechanism that triggers anti-inflammatory action. Curr Med Chem. 2009;16(19):2366–72. 10.2174/092986709788682119. PMID: 19601786. 19601786

[27] Auersperg V, Trieb K. Extracorporeal shock wave therapy: an update. EFORT Open Rev. 2020 Oct;5(10):584–92. 10.1302/2058-5241.5.190067. PMID: 33204500; PMCID: PMC7608508. PMC7608508 33204500

[28] Jeon SH, Zhu GQ, Kwon EB, Lee KW, Cho HJ, Ha US, et al. Extracorporeal shock wave therapy decreases COX-2 by inhibiting TLR4-NFkappaB pathway in a prostatitis rat model. Prostate. 2019 Sep;79(13):1498–504. 10.1002/pros.23880 31376214

[29] Santamato A, Beatrice R, Micello MF, Fortunato F, Panza F, Bristogiannis C, et al. Power doppler ultrasound findings before and after focused extracorporeal shock wave therapy for achilles tendinopathy: a pilot study on pain reduction and neovascularization effect. Ultrasound Med Biol. 2019 May;45(5):1316–23. 10.1016/j.ultrasmedbio.2018.12.009. Epub 2019 Feb 8. PMID: 30739723. 30739723

[30] Zimmermann R, Cumpanas A, Hoeltl L, Janetschek G, Stenzl A, Miclea F. Extracorporeal shock-wave therapy for treating chronic pelvic pain syndrome: a feasibility study and the first clinical results. BJU Int. 2008 Sep;102(8):976–80. 10.1111/j.1464-410X.2008.07742.x. Epub 2008 May 28. PMID: 18510660. 18510660

[31] Guu SJ, Geng JH, Chao IT, Lin HT, Lee YC, Juan YS, et al. Efficacy of low-intensity extracorporeal shock wave therapy on men with chronic pelvic pain syndrome refractory to 3-As therapy. Am J Mens Health. 2018 Mar;12(2):441–52. 10.1177/1557988317736585. Epub 2017 Oct 26 PMID: 29072124; PMCID: PMC5818120. PMC5818120 29072124

[32] Kong X, Hu W, Dong Z, Tian J, Wang Y, Jin C, et al. The efficacy and safety of low-intensity extracorporeal shock wave treatment combined with or without medications in chronic prostatitis/chronic pelvic pain syndrome: a systematic review and meta-analysis. Prostate Cancer Prostatic Dis. 2023;26:483–94. 10.1038/s41391-022-00571-0 35798855

[33] Li G, Man L. Low-intensity extracorporeal shock wave therapy for III B chronic pelvic pain syndrome. Transl Androl Urol. 2020 Jun;9(3):1323–8. 10.21037/tau.2020.04.07. PMID: 32676416; PMCID: PMC7354340. PMC7354340 32676416

[34] Al Edwan GM, Muheilan MM, Atta ON. Long term efficacy of extracorporeal shock wave therapy [ESWT] for treatment of refractory chronic abacterial prostatitis. Ann Med Surg (Lond). 2017 Jan;14:12–7. 10.1016/j.amsu.2016.12.051. PMID: 28119778; PMCID: PMC5237771. PMC5237771 28119778

[35] Mykoniatis I, Pyrgidis N, Sokolakis I, Sountoulides P, Hatzichristodoulou G, Apostolidis A, et al. Low-intensity shockwave therapy for the management of chronic prostatitis/chronic pelvic pain syndrome: a systematic review and meta-analysis. BJU Int. 2021 Aug;128(2):144–52. 10.1111/bju.15335. Epub 2021 Mar 3. PMID: 33434323. 33434323

